# A Role for STOML3 in Olfactory Sensory Transduction

**DOI:** 10.1523/ENEURO.0565-20.2021

**Published:** 2021-03-11

**Authors:** Emilio Agostinelli, Kevin Y. Gonzalez-Velandia, Andres Hernandez-Clavijo, Devendra Kumar Maurya, Elena Xerxa, Gary R. Lewin, Michele Dibattista, Anna Menini, Simone Pifferi

**Affiliations:** 1 Neurobiology Group, Scuola Internazionale Superiore di Studi Avanzati (SISSA), Trieste 34136, Italy; 2 Molecular Physiology of Somatic Sensation, Department of Neuroscience, Max Delbrück Center for Molecular Medicine, Berlin D-13122, Germany; 3 Department of Basic Medical Sciences, Neuroscience and Sensory Organs, University of Bari A. Moro, Bari 70124, Italy; 4 Department of Experimental and Clinical Medicine, Università Politecnica delle Marche, Ancona 60126, Italy

**Keywords:** ion channel, olfactory, transduction

## Abstract

Stomatin-like protein-3 (STOML3) is an integral membrane protein expressed in the cilia of olfactory sensory neurons (OSNs), but its functional role in this cell type has never been addressed. STOML3 is also expressed in dorsal root ganglia neurons, where it has been shown to be required for normal touch sensation. Here, we extended previous results indicating that STOML3 is mainly expressed in the knob and proximal cilia of OSNs. We additionally showed that mice lacking STOML3 have a morphologically normal olfactory epithelium. Because of its presence in the cilia, together with known olfactory transduction components, we hypothesized that STOML3 could be involved in modulating odorant responses in OSNs. To investigate the functional role of STOML3, we performed loose patch recordings from wild-type (WT) and *Stoml3* knock-out (KO) OSNs. We found that spontaneous mean firing activity was lower with additional shift in interspike intervals (ISIs) distributions in *Stoml3* KOs compared with WT neurons. Moreover, the firing activity in response to stimuli was reduced both in spike number and duration in neurons lacking STOML3 compared with WT neurons. Control experiments suggested that the primary deficit in neurons lacking STOML3 was at the level of transduction and not at the level of action potential generation. We conclude that STOML3 has a physiological role in olfaction, being required for normal sensory encoding by OSNs.

## Significance Statement

Olfactory transduction comprises a series of well-characterized molecular steps that take place in the cilia of olfactory sensory neurons (OSNs) terminating in action potential firing. Here, we introduce a possible new player: stomatin-like protein-3 (STOML3). Indeed, STOML3 is localized in olfactory cilia, and we show that STOML3 plays a role in OSN physiology. First, it allows OSNs to broaden the possible frequency range of their spontaneous activity. Second, STOML3 modulates odorant-evoked action potential firing by regulating both the number of spikes and response duration. These new findings call for a reconsideration of the patterns of the peripheral coding of sensory stimuli.

## Introduction

Olfactory perception begins with the binding of molecules to odorant receptors in the cilia of olfactory sensory neurons (OSNs) in the olfactory epithelium. This binding initiates a transduction cascade that involves the activation of G-proteins, adenylyl cyclase Type III (ACIII), phosphodiesterases (PDEs), CNG, and TMEM16B ion channels (Jones and Reed, 1989; Buck and Axel, 1991; Brunet et al., 1996; Wong et al., 2000; Cygnar and Zhao, 2009; Pifferi et al., 2010; Billig et al., 2011; Reisert and Reingruber, 2019). The activation of the transduction cascade leads to the generation of inward currents that produce membrane depolarization, the activation of voltage-gated currents and the generation of action potentials that propagate along the axons of OSNs to the olfactory bulb (Schild and Restrepo, 1998; Firestein, 2001; Mombaerts, 2004; Kleene, 2008; Dibattista et al., 2017).

The physiological role of several proteins highly expressed in the cilia of OSNs, including stomatin-like protein-3 (STOML3), is still unknown. STOML3 was identified in the mouse olfactory epithelium by Kobayakawa et al. (2002), who first named it SRO, and one year later by Goldstein et al. (2003), who named it SLP3. STOML3 is a member of the family of stomatin-like proteins characterized by the presence of a structurally conserved core domain of nearly 120 residues called the stomatin domain, which is further related to the SPFH domain (Stomatin, Prohibitin, Flotillin, HflK/HflC; Tavernarakis et al., 1999; Green and Young, 2008; Lapatsina et al., 2012). Stomatin-like proteins are found in all three domains of life and show remarkable conservation, with bacterial and human homologs sharing 50% identity. In the mammalian genome, 5 members of the stomatin family have been identified with 40–84% sequence similarity in the stomatin domain and with similar membrane topology characterized by a single, relatively short, hydrophobic membrane insertion domain, followed by the core stomatin domain (Green and Young, 2008; Lapatsina et al., 2012). Several recent studies have revealed some common aspects of the physiology of stomatin proteins. In particular, it has been demonstrated that they can form oligomers, mostly localize to membrane domains and modulate ion channel activity, even if the precise mechanisms of this regulation are still unclear (Brand et al., 2012; Lapatsina et al., 2012; Poole et al., 2014; Wetzel et al., 2017; Klipp et al., 2020).

As mentioned above, STOML3 was first shown to be expressed in OSNs (Kobayakawa et al., 2002; Goldstein et al., 2003; Kulaga et al., 2004; Tadenev et al., 2011), and we hypothesized that it might play a role in the sensory response to odorants. In this study, we first analyzed in detail the expression and subcellular localization of STOML3 in the mouse olfactory epithelium and confirmed its presence in the cilia of OSNs, the site of olfactory transduction. By using a *Stoml3* knock-out (KO) mouse line (Wetzel et al., 2007), we first verified that mice lacking STOML3 do not present alterations in olfactory epithelium morphology and show similar expression levels of proteins involved in transduction as wild-type (WT) mice. We then asked whether STOML3 plays a role in OSN stimulus coding by measuring spontaneous and stimulus-induced spiking activity in the loose-patch configuration in WT and *Stoml3* KO mice. We found that STOML3 regulates both spontaneous firing and responses to 3-isobutyl-1-methylxanthine (IBMX) and odorant mixtures, and we discuss several possible scenarios for the role that STOML3 might play in olfactory transduction.

## Materials and Methods

### Animals

Mice were handled in accordance with the Italian Guidelines for the Use of Laboratory Animals and the European Union guidelines on animal research according to a protocol approved by the ethics committee Scuola Internazionale Superiore di Studi Avanzati (SISSA). Experiments were performed on tissues from C57BL/6 WT and *Stoml3* KO mice of either sex (Wetzel et al., 2007).

### Immunohistochemistry

The head containing the nasal cavity was fixed in 4% paraformaldehyde in PBS at pH 7.4 for 4 h at 4°C. After fixing, the heads of the mice were incubated in 0.5 m EDTA for 2 d. The tissues were cryoprotected by incubation in 30% sucrose in PBS at pH 7.4 overnight. The tissue was immersed in cryostat embedding medium (BioOptica) and immediately frozen at −80°C. Coronal sections (16–18 μm) were cut on a cryostat and mounted on Superfrost Plus Adhesion Microscope Slides (ThermoFisher Scientific). The sections were air-dried for 3 h. To wash the cryostat embedding medium from tissue, the slices were incubated for 15 min with PBS. The tissue was treated for 15 min with 0.5% SDS in PBS for antigen retrieval, washed and incubated in blocking solution (2% normal donkey/calf serum, 0.2% Triton X-100 in PBS) for 90 min and finally in primary antibodies diluted in blocking solution overnight at 4°C. The following primary antibodies (catalog number, dilution; company) were used: mouse monoclonal anti-acetylated tubulin (T7451; 1:100; Sigma), polyclonal rabbit anti-ACIII (sc-588; 1:100, Santa Cruz Biotechnology), polyclonal goat anti-CNGA2 (sc-13700; 1:100, Santa Cruz Biotechnology), polyclonal rabbit anti-Ki67 (sc-7846, 1:250, Santa Cruz Biotechnology), polyclonal goat anti-OMP (544-10001, 1:1000, Wako), mouse monoclonal anti-p63 (CM163, 1:250, Biocare Medical), polyclonal rabbit anti-STOML3 (13316-1-AP, 1:200, Proteintech), and polyclonal rabbit anti-TMEM16B (NBP1-90739,1:250, Novus). After the removal of the excess primary antibodies with PBS washes, the sections were incubated with Alexa Fluor-conjugated secondary antibodies (1:500 dilution) in TPBS (0.2% Tween 20 in PBS) for 2 h at room temperature, washed and mounted with Vectashield (Vector Laboratories) or FluoromontG (ThermoFisher). DAPI (5 μg/ml) was added to the solution containing secondary antibody to stain the nuclei. The secondary antibodies used were Alexa Fluor 594-conjugated donkey anti-goat, Alexa Fluor 594-conjugated chicken anti-rabbit, Alexa Fluor 488-conjugated goat anti-rabbit, and Alexa Fluor 488-conjugated donkey anti-mouse (ThermoFisher). For the anti-Ki67 and anti-p63 antibodies, we used a heat antigen retrieval protocol instead of treatment with SDS. The tissue was put in a container with sodium citrate buffer (10 mm sodium citrate, 0.05% Tween 20, pH 6.0) and heated at 100°C in a microwave for 5 min. After cooling, the sodium citrate buffer was washed, and the rest of the procedure was the same as aforementioned. To reveal anti-STOML3, we applied the tyramide signal amplification method using the Tyramide SuperBoost kit (ThermoFisher; Hunyady et al., 1996). Immunoreactivity was visualized with a confocal microscope (Nikon A1R or C1). Images were acquired using NIS Element software (Nikon) at 1024 × 1024-pixel resolution and were not modified other than to balance brightness and contrast.

### Cell counting

To quantify OMP-immunopositive, Ki67-immunopositive, and p63-immunopositive cells, we used the ImageJ cell counter tool. Cell density was estimated by counting the immunopositive cells in a 100 × 100 μm^2^ area. Approximately 18–20 areas, spanning several widely separated regions in the olfactory epithelium from the anterior part of Turbinate II to the ventral portion of Turbinate IV, were selected for counting from each mouse. Three mice from each genotype were used, and nine coronal sections with a thickness of 16 μm were collected from each animal.

### Whole-mount cilia staining

The olfactory turbinates were exposed by bisecting the head along the midline and removing the septum. The hemiheads were fixed with 4% paraformaldehyde in PBS at pH 7.4 for 20 min at room temperature and then washed with PBS. Endogenous biotin was blocked by using an Avidin/Biotin Blocking kit (Vector Laboratories) following the manufacturer’s protocol. The preparation was incubated for 2 h with the biotinylated lectin *Dolichos biflorus* agglutinin (DBA; VectorLabs; Lipscomb et al., 2002) at a concentration of 20 μg/ml in PBS and then washed three times with PBS for 10 min each. The preparation was incubated for 3 h with streptavidin-Alexa Fluor 594 (ThermoFisher) diluted 1:500 in PBS and washed again. Then, the turbinates were dissected from the nasal cavity and mounted on FluoroDishes (World Precision Instruments) with Vectashield (Vector Laboratories) or Fluoromont-G (ThermoFisher). A coverslip was gently placed on the tissue to press and close the cilia to the glass bottom of the FluoroDish. Cilia length was measured using Fiji software (Schindelin et al., 2012) using the segmented line tool on Z-projection images.

### Western blotting

Olfactory epithelium from adult mice was lysed with RIPA buffer (25 mm Tris at pH 7.5, 1% NP40, 150 mm NaCl, 0.5% deoxycholate, and 0.1% SDS) containing Complete Mini Protease Inhibitor Cocktail (Roche). A total of 300 μl of lysis buffer was used for each mouse. The tissue was homogenized with an electrical homogenizer at low speed. After lysis, the homogenized tissue was incubated for 1 h on ice and mixed gently every 15 min. The lysate was cleared by centrifugation for 10 min at 10,000 × *g* at 4°C. The supernatant was stored separately, and its protein concentration was measured with a Pierce BCA Protein Assay kit (ThermoScientific) according to the manufacturer’s protocol.

For Western blotting, 40 μg of protein extract dissolved in Laemmli buffer (2× buffer concentration: 125 mm Tris at pH 6.8, 4% SDS, 20% glycerol, 0.1% bromophenol blue, and 10% β-mercaptoethanol added fresh) was loaded in each well of an 8–12% acrylamide/bis-acrylamide gel (Sigma) for SDS-PAGE electrophoresis. After separation, the gel containing the proteins was equilibrated for 10 min in transfer buffer (25 mm Tris, 192 mm glycine pH 8.3, and 15% MeOH), and then the proteins were transferred to PVDF membranes with a pore size of 0.45 μm (ThermoScientific). The membrane was blocked in TBS with 5% nonfat dried milk at room temperature for 1 h and then incubated overnight with primary antibodies diluted in 0.1% Tween 20 in TBS (TBS-T). Then, the membrane was washed three times with TBS-T and incubated with horseradish peroxidase-conjugated secondary antibodies in TBS-T for 1 h. The signal was developed using Super Signal West Pico PLUS Chemiluminescent substrate (ThermoScientific) or Super Signal West Atto Ultimate Chemiluminescent substrate (ThermoScientific). The following primary antibodies (catalog number, dilution; company) were used: polyclonal rabbit anti-ACIII (sc-588; 1:1000, Santa Cruz Biotechnology), polyclonal rabbit anti-CNGA2 (ab96410; 1:500, Abcam), polyclonal goat anti-OMP (544-10001, 1:5000, Wako), polyclonal rabbit anti-STOML-3 (13316-1-AP, 1:1000, Proteintech), polyclonal rabbit anti-TMEM16B (20647-1-AP, 1:2000, Proteintech), and monoclonal mouse anti-α-tubulin (T8203, 1:5000, Sigma). The following secondary antibodies (catalog number, dilution; company) were used: polyclonal goat anti-rabbit horseradish peroxidase (HRP; P0448, 1:2000, Dako), polyclonal rabbit anti-goat HRP (P0449, 1:2000, Dako), and polyclonal goat anti-mouse HRP (P0447, 1:2000, Dako). Proteins were not subjected to any deglycosylation protocol and were identified according to their expected molecular size: STOML3 (∼30 kDa), TMEM16B (∼170 kDa), OMP (∼22 kDa), ACIII (∼250 kDa), CNG (∼120 kDa), and α-tubulin (∼55 kDa). For STOML3 ANO2 and OMP, the size was additionally confirmed by comparing the bands in WT and *Stoml3*, *Tmem16b*, or *Omp* KO models (Potter et al., 2001; Wetzel et al., 2007; Zhang et al., 2017).

### Electrophysiological recordings from OSNs

Acute coronal slices of the olfactory epithelium were obtained from P0-P4 mice using a method similar to previously described methods (Shimazaki et al., 2006; Dibattista et al., 2008; Pietra et al., 2016; Wong et al., 2018; Henriques et al., 2019). The head of a P0-P4 mouse without the skin was dissected and embedded in 3% Type I-A agarose prepared in Ringer’s solution once the solution cooled to 38°C. Ringer’s solution contained 140 mm NaCl, 5 mm KCl, 2 mm CaCl_2_, 1 mm MgCl_2_, 10 mm HEPES, and 10 mm glucose and was adjusted to pH 7.4 with NaOH. Coronal slices from the mouse olfactory epithelium with a thickness of 300 μm were cut with a vibratome (Vibratome 1000 Plus Sectioning System) and kept in cold oxygenated Ringer’s solution until use. Postnatal day (P)0–P4 mice are suitable for this study because STOML3 is expressed beginning as early as the embryonic stage embryonic day (E)17.5 (Goldstein et al., 2003).

Slices were transferred to a recording chamber continuously perfused with oxygenated Ringer’s solution at room temperature and viewed with an upright microscope (BX51WI; Olympus) equipped with infrared differential contrast optics, a camera (DFK 72BUC02; Imaging Source) and a 40 × water-immersion objective with an additional 2× auxiliary lens. OSNs were identified by their morphology. The recordings were performed at room temperature (20–25°C) using a MultiClamp 700B amplifier controlled by Clampex 10.6 via a Digidata 1550B (Molecular Devices). Data were low-pass filtered at 2 kHz and sampled at 10 kHz. The bath was ground via a 3 m KCl agar bridge connected to an Ag/AgCl reference electrode. Patch pipettes were pulled from borosilicate capillaries (WPI) with a Narishige PC-10 puller.

Extracellular recordings from the soma of OSNs were obtained in the on-cell loose-patch configuration using patch pipettes with a resistance of 2–3 MΩ when filled with Ringer solution. The seal resistances were 30–50 MΩ. Extracellular recordings were made in voltage-clamp mode with a holding potential of 0 mV. Stimuli were delivered through an eight-into-one multibarrel perfusion pencil connected to a ValveLink8.2 pinch valve perfusion system (Automate Scientific). To test cell viability, a high K^+^ Ringer solution (25 mm KCl) was applied in 3-s pulses, and experiments were performed only on OSNs responding to this stimulus.

Control experiments measuring membrane properties and inward and outward voltage-gated currents were performed in the whole-cell voltage-clamp configuration. Patch pipettes had resistances of 4–7 MΩ when filled with the intracellular solution composed of 145 mm KCl, 4 mm MgCl_2_, 0.5 mm EGTA, and 10 mm HEPES, adjusted to pH 7.2 with KOH.

Heptaldehyde, isoamyl acetate, acetophenol, cineole and eugenol were dissolved in dimethyl sulfoxide (DMSO) at 5 m to generate stock solutions. The odorant mixture was prepared by diluting each odorant at a final concentration of 100 μm on the day of the experiment. This concentration was chosen because it elicits saturating responses from OSNs expressing several types of odorant receptors (Dibattista and Reisert, 2016). IBMX was prepared weekly by dissolving in Ringer’s solution at a final concentration of 1 mm. At this concentration, IBMX elicits responses with kinetics similar to those induced by odorants (Reisert et al., 2007). All chemicals were purchased from Sigma unless otherwise specified.

### Data analysis

IgorPro software (WaveMetrics) and Clampfit (Molecular Devices) were used for data analysis and figure preparation.

Recordings were filtered offline with a high-pass filter at 2 Hz to eliminate slow drifts in the baseline. Individual action potentials were identified by an event detection algorithm using an arbitrary threshold, and each event was confirmed by shape inspection. The starting time of each event was taken as the time for that individual action potential. The mean spontaneous firing frequency was calculated as the number of spikes divided by the duration of the recording. The interspike interval (ISI) was calculated by measuring the time between consecutive spikes (second to first, third to second, and so forth). To construct the ISI distribution, we calculated the ISI for all spikes for each cell. Then, we grouped the ISIs in bins as indicated in the figures and divided the value of each bin by the total number of calculated ISIs for each cell. Finally, we averaged the distribution obtained from all cells of the group (Arnson and Holy, 2011). The values in the *y*-axes represent the percentage of spikes in each bin, and the area under the curve is 100%.

The averages obtained from individual experiments in different cells are presented as the mean ± SEM and the number of cells recorded (*n*). Cells were obtained from at least three different WT or KO mice. Since some data were not normally distributed (Jarque–Bera test or Shapiro–Wilk test), statistical significance was determined by Wilcoxon–Mann–Whitney’s test (*U* test). The Kolmogorov–Smirnov test was used to compare the cumulative distributions; *p* < 0.05 were considered statistically significant.

## Results

### STOML3 expression in the olfactory epithelium

The STOML3 protein has been shown to be expressed in the mouse olfactory epithelium and to localize in mature OSNs (Kobayakawa et al., 2002; Goldstein et al., 2003; Kulaga et al., 2004; Tadenev et al., 2011), but its functional role in the olfactory system has not been investigated. We examined the role of the STOML3 protein in OSNs by taking advantage of a loss-of-function approach using the *Stoml3* KO mouse model.

**Figure 1.**
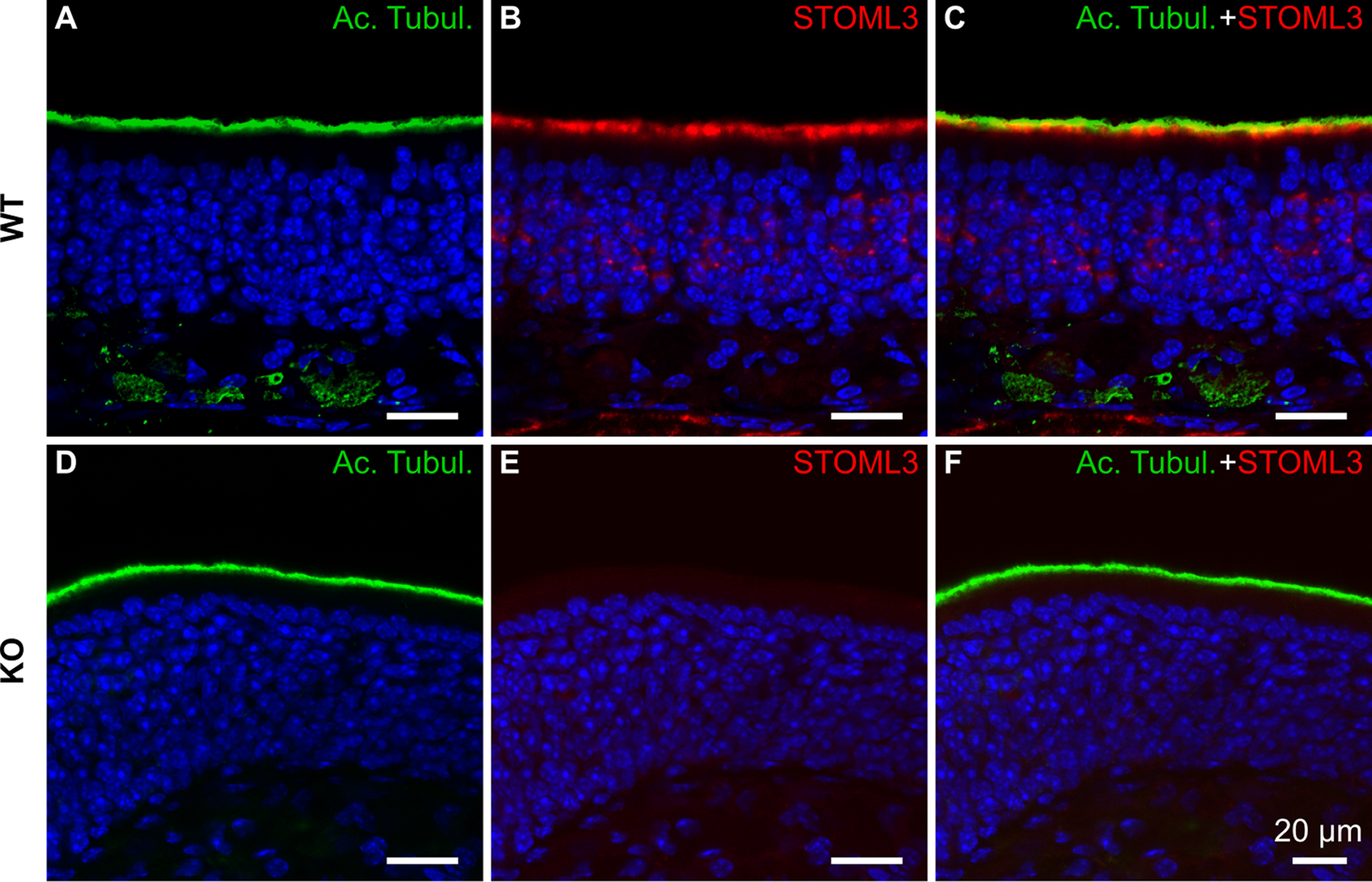
STOML3 is expressed in knob/proximal cilia of OSNs. ***A***, ***D***, Confocal micrographs of coronal sections of the olfactory epithelium from WT and KO mice immunostained with antibody against the ciliary marker acetylated tubulin (Ac. Tubul.). ***B***, ***E***, Immunostaining for STOML3 from WT and KO mice. ***C*,** Merging of the signals shows that STOML3 has diffuse staining that colocalizes with acetylated tubulin (yellow regions in ***C***) and more defined staining of the layer below the Ac. Tubul. in the region occupied by the OSN knobs. The signal disappeared in the olfactory epithelium of the *Stoml3* KO mice (***E***, ***F***). Nuclei were stained with DAPI (blue).

First, we sought to confirm the expression pattern of STOML3 in the olfactory epithelium of WT mice with immunofluorescence. Immunostaining for STOML3 showed clear staining of the ciliary layer (Fig. 1). In particular, the staining pattern showed the localization of STOML3 in the knob and proximal parts of the cilia of OSNs (Fig. 1*A–C*). Cilia were visualized by marking the ciliary protein acetylated tubulin, and we observed a partial overlap with STOML3 (Fig. 1*C*). While the acetylated tubulin pattern seemed unaltered in KO mice, STOML3 staining was absent, thus demonstrating the specificity of our staining (Fig. 1*D–F*). In addition to the intense signal of the knob/cilia, we also observed sparse and punctate localization of STOML3 in the mature OSN cell bodies, which also disappeared in the KO mice (Fig. 1*B*, *E*).

Together, our data confirm and extend previous results showing that STOML3 is mainly expressed in the knob and proximal ciliary regions of OSNs.

### *Stoml3* gene deletion does not alter the structural and morphologic properties of the olfactory epithelium

As stomatin proteins have been implicated in the structural and morphologic organization of different cellular complexes (Kobayakawa et al., 2002; Umlauf et al., 2006; Browman et al., 2007; Wetzel et al., 2007; Brand et al., 2012; Lapatsina et al., 2012; Poole et al., 2014; Qi et al., 2015), we asked whether STOML3 is important for the structural organization of the olfactory epithelium and its cell types.

**Figure 2.**
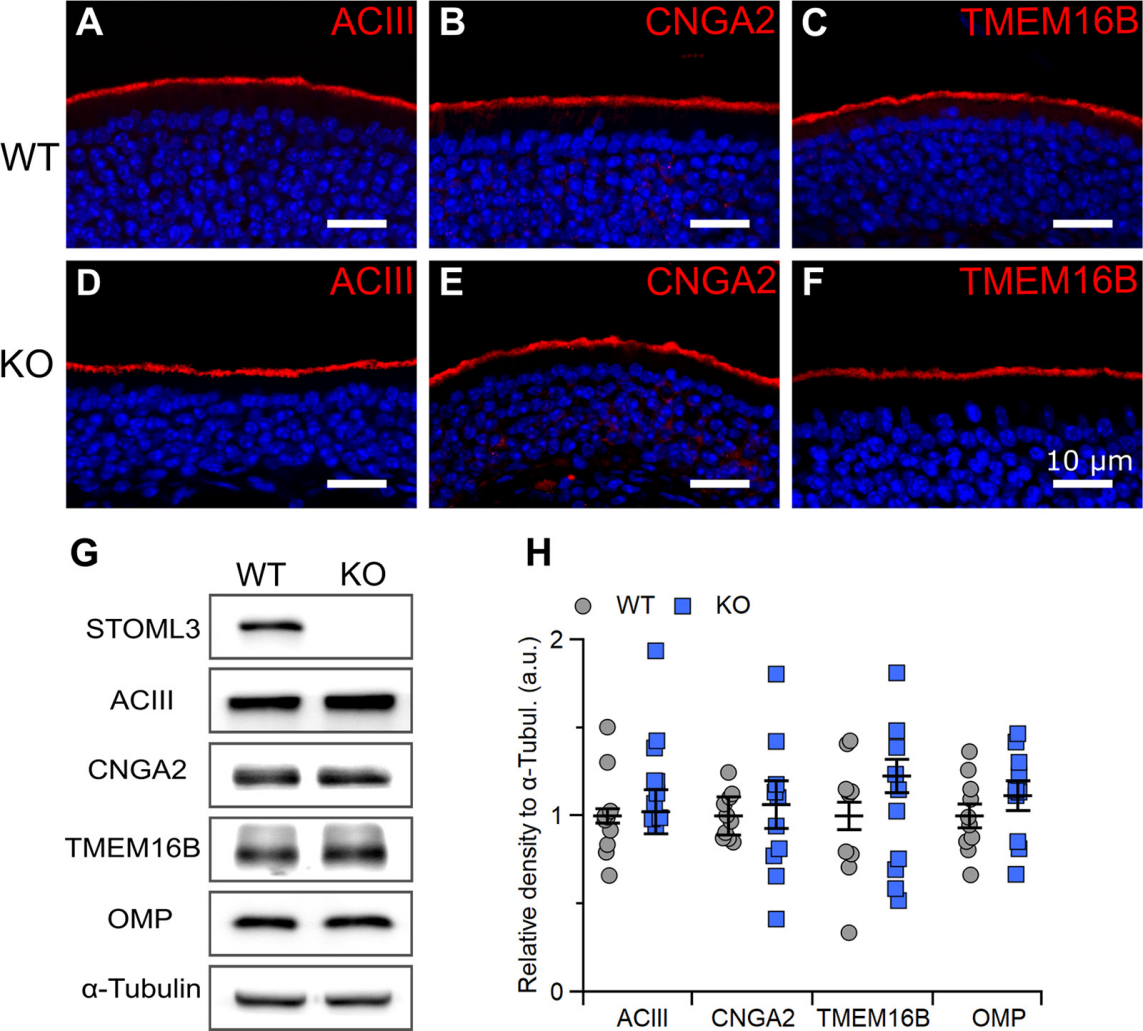
*Stoml3* KO does not alter the localization or expression level of molecular components of olfactory transduction. ***A–F***, Confocal micrographs of coronal sections of the olfactory epithelium from WT and KO mice immunostained for ACIII, CNGA2, or TMEM16B. Nuclei were stained with DAPI (blue). ***G***, Western blot analysis of olfactory epithelium proteins for STOML3, ACIII, CNGA2, TMEM16B, OMP, and α-tubulin. The specific 32-kDa band for STOML3 is missing in proteins from KO mice. ***H***, Expression levels relative to α-tubulin for the indicated proteins in olfactory epithelium from 10 WT and KO mice.

We performed immunostaining for the signal transduction machinery proteins that are abundantly expressed in OSN cilia and found that ACIII, CNGA2, and TMEM16B localized to the ciliary layer in both WT and *Stoml3* KO mice (Fig. 2*A–F*). Moreover, while keeping the same acquisition settings, we did not observe any clear differences in the staining intensity or in the expression pattern in KO versus WT olfactory epithelia.

Then, we performed Western blot analysis to assess changes in the expression levels of signal transduction machinery proteins. Using an antibody against STOML3, we identified a 32-kDa band in WT mice corresponding to the expected molecular weight of the STOML3 protein that was absent in KO mice, while bands corresponding to ACIII, CNGA2, TMEM16B, OMP, and acetylated tubulin were present in both mouse lines (Fig. 2*G*). The protein expression levels normalized to α-tubulin were similar in WT and KO mice (Fig. 2*H*).

Taken together, these results indicate that STOML3 is not involved in regulating the localization or expression levels of several members of the olfactory transduction cascade.

**Figure 3.**
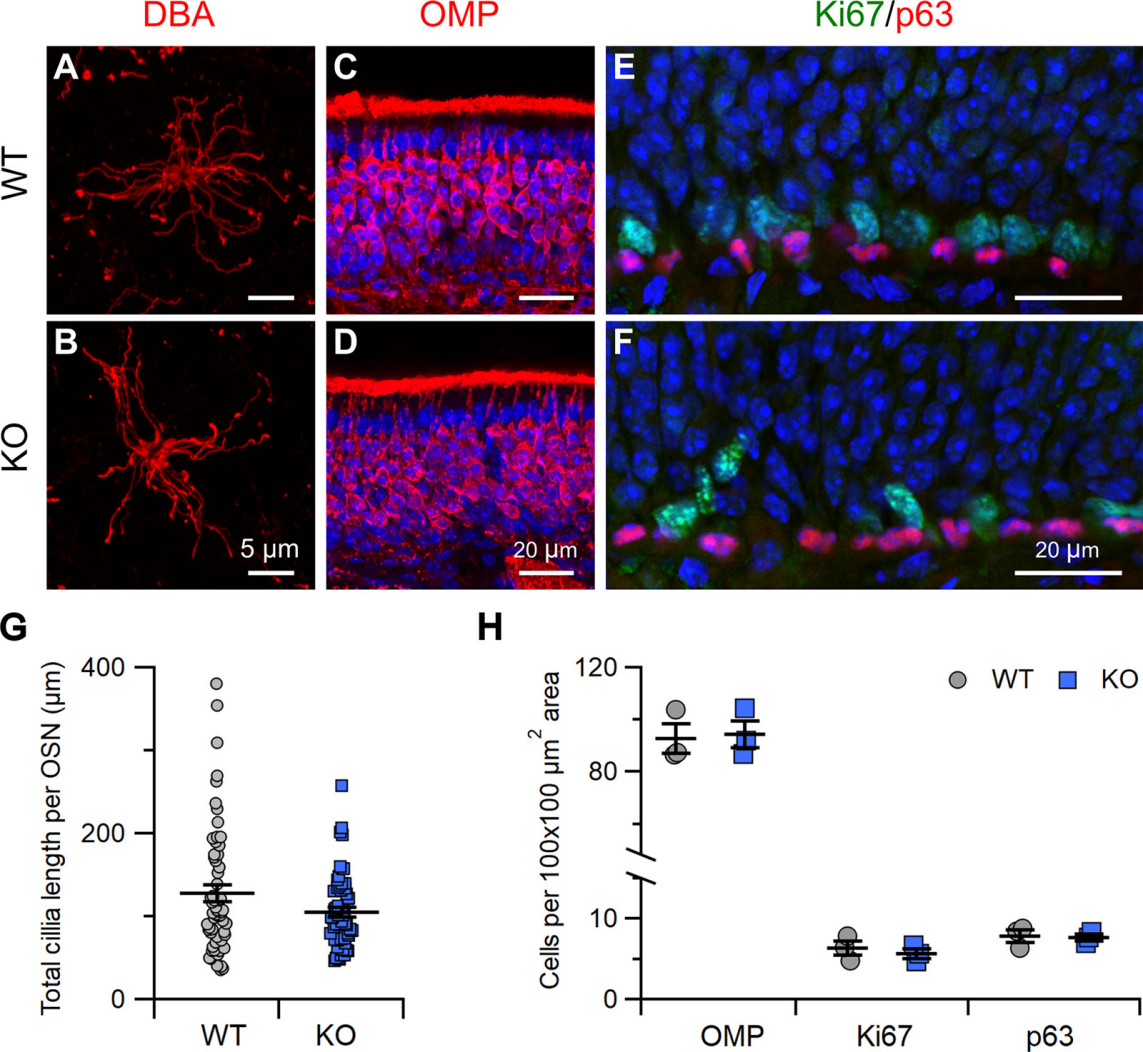
*Stoml3* KO mice have grossly normal olfactory epithelium. ***A***, ***B***, Enface view of a whole-mount preparation of olfactory epithelium with OSN cilia labeled by biotinylated DBA detected by streptavidin-Alexa Fluor 594. ***C–F***, Confocal micrographs of coronal sections of the olfactory epithelium from WT and KO mice immunostained for OMP (***C***, ***D***) or Ki67 and p63 (***E***, ***F***). Nuclei were stained with DAPI (blue). ***G***, Quantification of the total cilia length per OSN from WT and KO mice (OSNs: *n* = 61 from 4 WT mice, *n* = 57 from 4 KO mice). ***H***, Quantification of OMP-immunopositive, Ki67-immunopositive, and p63-immunopositive cells in olfactory epithelium from WT and KO mice.

To better assess whether STOML3 could have a role in neuronal morphologic organization, we used DBA to mark the entire length of OSN cilia. We quantified the total ciliary length per OSN and determined that although there was a tendency for the KO to have a lower total ciliary length, the difference was not significant (Fig. 3*A*,*B*; quantification in Fig. 3*G*; *p* = 0.23 *U* test). Additionally, the overall olfactory epithelium morphology was not changed since the staining and counting of OMP-positive cells (Fig. 3*C*,*D*) did not reveal any difference in the density of OMP-positive cells between WT and KO mice (92 ± 5 for WT and 94 ± 5 for KO, cells per 100 × 100 μm^2^ area; Fig. 3*H*).

To further characterize the morphology of the olfactory epithelium, we stained the population of basal cells with Ki67 and p63, to stain globose basal cells (GBCs) and horizontal basal cells (HBCs), respectively. These cells are usually located at the base of the olfactory epithelium lying on the basal lamina. We observed Ki67-positive and p63-positive cells in their expected locations in both WT and KO mice (Fig. 3*E*,*F*). In addition, no differences were observed in the density of HBC and GBC cells (Ki67: 6.3 ± 0.9 for WT and 5.6 ± 0.6 for KO; p63: 7.8 ± 0.8 for WT and 7.7 ± 0.4 for KO; cells per 100 × 100 μm^2^ area; Fig. 3*H*).

In summary, the absence of STOML3 did not alter the density of OSNs, HBCs or GBCs, indicating that STOML3 is not involved in normal olfactory epithelium development.

### STOML3 modulates spontaneous firing frequency in OSNs

Based on recent findings showing that STOML3 contributes to shaping the electrophysiological properties of skin mechanoreceptors (Wetzel et al., 2007, 2017; Poole et al., 2014; Qi et al., 2015) and on its expression in OSNs, we hypothesized that STOML3 might play a role in the spontaneous and/or evoked electrical activities of OSNs.

We first tested whether STOML3 deletion modifies OSN passive membrane properties performing whole-cell recordings on olfactory epithelium slices from P0-P4 mice with KCl in the patch pipette. Both resting membrane potential (−55 ± 1 mV, *n* = 48 for WT, and −51 ± 1 mV, *n* = 41 for KO, *p* > 0.05 *U* test), and input resistance (2.5 ± 0.3 GΩ, *n* = 48 for WT, and 2.4 ± 0.3 GΩ, *n* = 41 for KO, *p* > 0.05 *U* test) were not significantly different between OSNs from WT and *Stoml3* KO mice. Also, we did not find significant differences in the amplitude of inward (−673 ± 69 pA, *n* = 48 for WT, and −626 ± 58 pA, *n* = 41 for KO, *p* > 0.05 *U* test), and outward voltage-gated currents (1425 ± 96 pA, *n* = 48 for WT, and 1635 ± 139 pA, *n* = 41 for KO, *p* > 0.05 *U* test).

**Figure 4.**
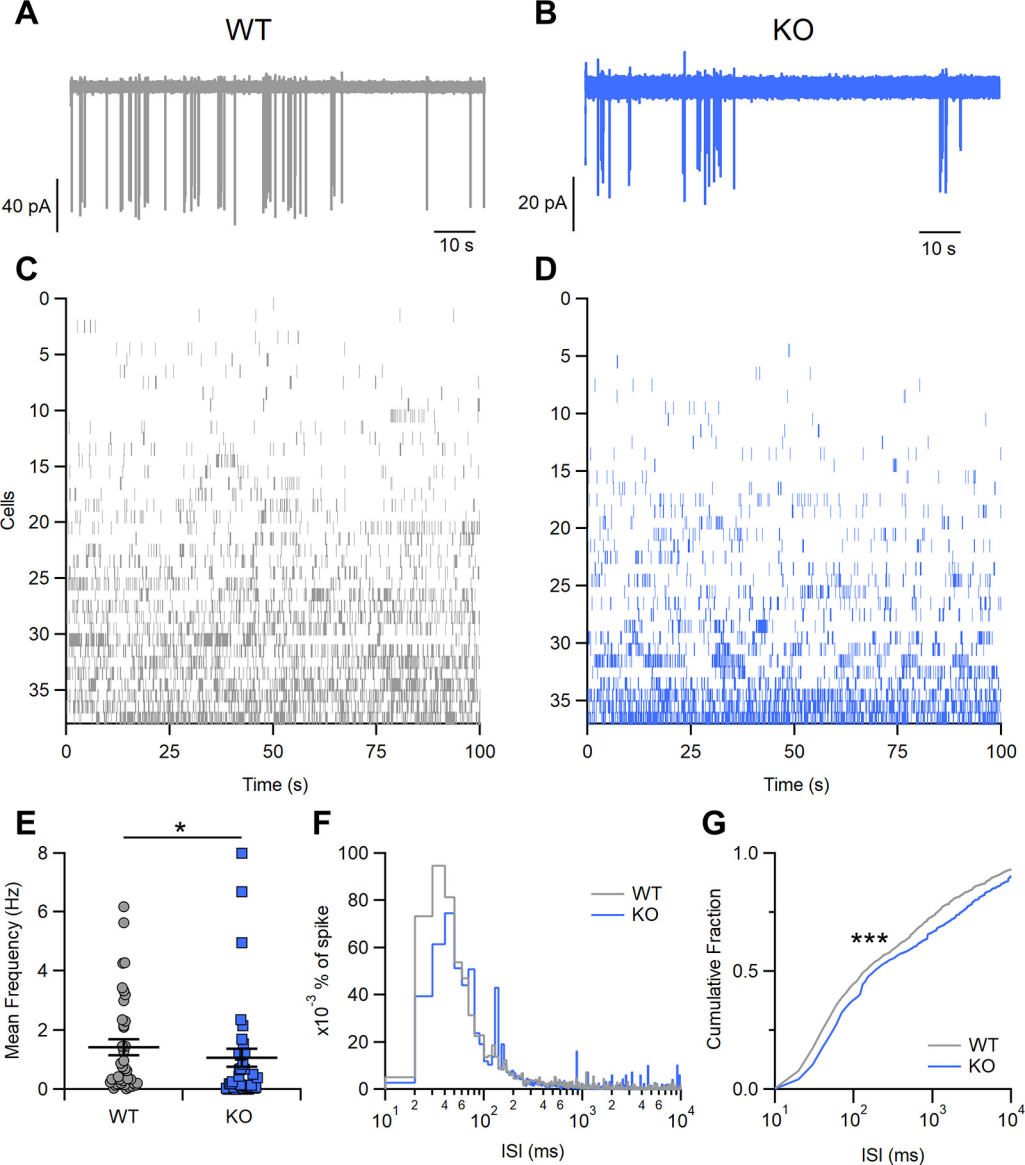
Spontaneous firing activity in OSNs in WT and *Stoml3* KO mice ***A***, ***B***, Representative loose-patch recordings showing the spontaneous activity of OSNs from acute slices of olfactory epithelium from WT and KO mice. ***C***, ***D***, Raster plots of recordings of spontaneous activity from several OSNs from WT and KO mice. Each row represents spike activity from a different OSN. ***E***, Mean frequency of spontaneous activity in OSNs from WT and KO mice (*n* = 38 for WT and *n* = 37 for KO, *p < 0.05 *U* test). ***F***, ISI distributions of spontaneous firing from cells shown in ***C***, ***D*** (bin = 5 ms). Values were normalized to the area under each curve to show the spike percentages for WT or KO mice. ***G***, Cumulative fraction of ISI distributions (***p < 0.001, Kolmogorov–Smirnov test).

We then used loose patch recordings to investigate the spontaneous activity of OSNs in olfactory epithelium slices from WT and *Stoml3* KO mice. By recording from a substantial number of cells, we could appreciate the heterogeneous patterns of spontaneous activity in WT OSNs (Fig. 4*A*,*C*). The raster plots of 38 WT cells showed a very large variation in firing patterns ranging from almost silent OSNs (Fig. 4*C*, upper rows) to OSNs with elevated and sustained spontaneous firing (Fig. 4*C*, bottom rows). Both variance of firing rates and ISIs in WT OSNs were similar to those previously reported (Reisert, 2010; Connelly et al., 2013; Lorenzon et al., 2015; Pietra et al., 2016). In *Stoml3* KO mice, OSNs also displayed large variations in firing patterns, and the raster plots (Fig. 4*D*) indicate that there was an increased number of OSNs with lower spontaneous firing compared with WT controls. Indeed, OSNs in KO had a lower spontaneous mean frequency (1.1 ± 0.3 Hz, *n* = 37) than those in WT mice (1.4 ± 0.3 Hz, *n* = 38; *p* < 0.05 *U* test; Fig. 4*E*).

In addition, the inter spike interval (ISI) distribution revealed that short ISIs were much reduced in *Stoml3* KO neurons compared with WT neurons (Fig. 4*F*). The overall cumulative probability further showed that the ISI distribution was different between the WT and KO neurons (*p* < 0.001 Kolmogorov–Smirnov test; Fig. 4*G*).

These results demonstrate that STOML3 plays a role in shaping OSN spontaneous activity. Indeed, in the absence of STOML3, there was a decrease in the mean firing frequency and an increase in the ISIs of spontaneous activity compared with WT OSNs.

### STOML3 regulates the spike number and duration of the evoked response

As spontaneous firing in OSNs is mainly driven by the constitutive activity of the OR that initiates and controls the activation of the downstream signaling cascade (Reisert, 2010; Connelly et al., 2013; Dibattista and Reisert, 2016), the changes in the spontaneous firing of OSNs lacking STOML3 led us to hypothesize that STOML3 might play a role in odorant signal transduction and the electrical response to odorants.

To further understand the potential involvement of STOML3 in transduction events, we recorded action potential firing in response to the PDE inhibitor IBMX, often used as an odorant mimic, as blocking PDE reveals the basal rate of cAMP production by ACIII (Reisert, 2010; Dibattista and Reisert, 2016; Pietra et al., 2016).

**Figure 5.**
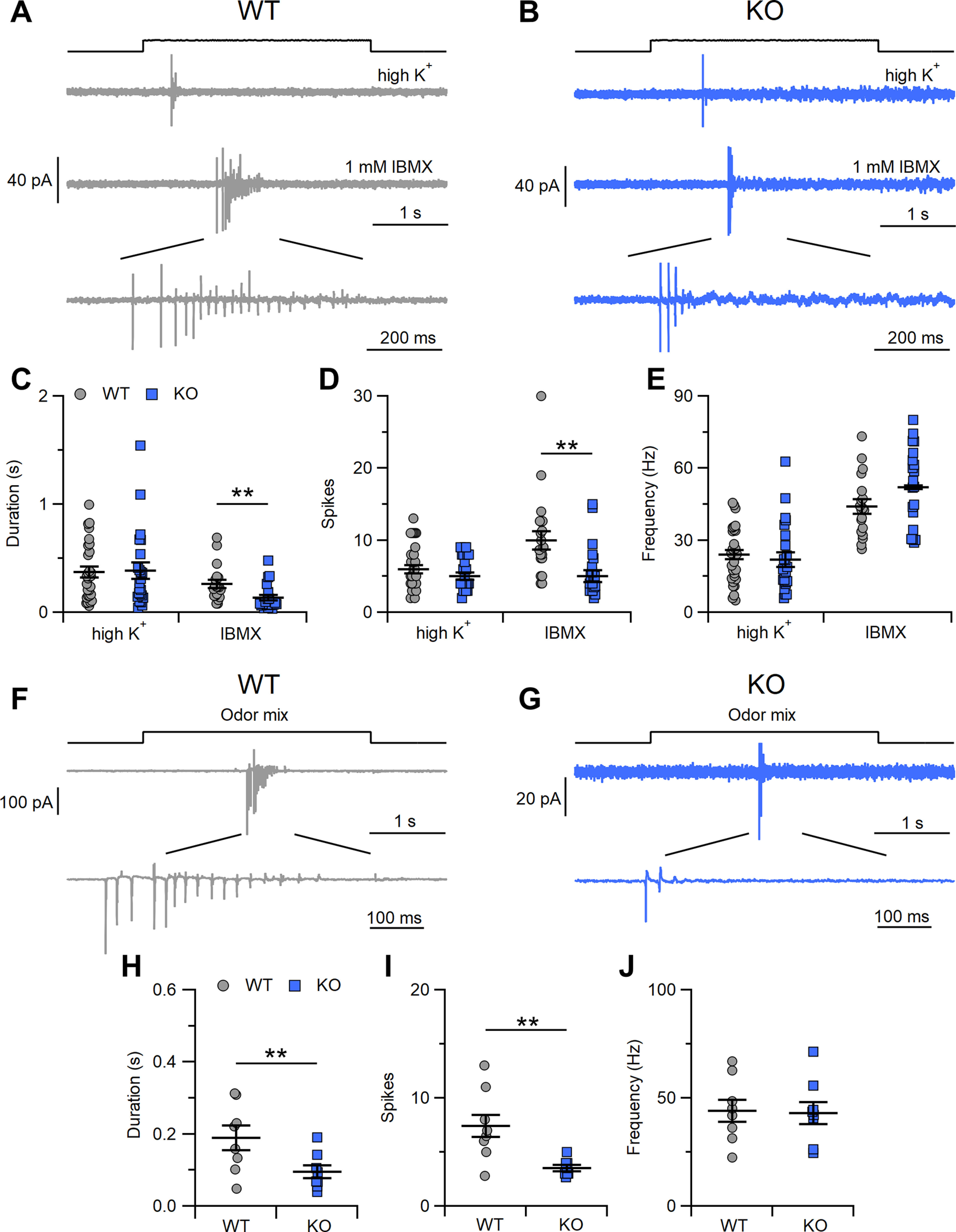
Evoked activity in OSNs in WT and *Stoml3* KO mice. ***A***, ***B***, Representative loose-patch recordings of OSNs from WT and KO mice stimulated with high K^+^ (upper trace) or with 1 mm IBMX (middle and lower traces). Comparison of response duration (***C***), number of evoked spikes (***D***), and mean frequency of the response (***E***) to high K^+^ or 1 mm IBMX in OSNs from WT and KO mice (*n* = 19 for WT, *n* = 20 for KO; **p < 0.01 *U* test). ***F***, ***G***, Representative loose-patch recordings of OSNs from WT and KO mice stimulated with odor mixture. Comparison of response duration (***H***), number of evoked spikes (***I***), and mean frequency of the response (***J***) to odor mixture in OSNs from WT and KO mice (*n* = 8 for WT and KO; **p < 0.01 *U* test).

In the loose patch configuration, we stimulated OSNs for 3 s with 1 mm IBMX and recorded the evoked firing activity (Fig. 5). A depolarizing stimulus made by a high K^+^ solution was also applied to measure action potential firing activity independent of the activation of the transduction cascade by IBMX. The average duration and number of spikes in response to high K^+^ were similar in WT (372 ± 49 ms, 6.0 ± 0.6) and KO (385 ± 75 ms, 5.0 ± 0.5) mice (*p* > 0.05 *U* test; Fig. 5*C*,*D*). However, responses to IBMX differed between the genotypes. Indeed, although the mean firing frequency of the response to IBMX was on average 50 Hz in both WT and KO OSNs (Fig. 5*E*), the number of evoked spikes was substantially lower (5 ± 1) in KO than in WT (10 ± 1) mice (*p* < 0.01 *U* test), and the duration of the response was shorter in KO (135 ± 27 ms) than in WT (263 ± 38 ms) mice (*p* < 0.01 *U* test). These differences were because of changes in the transduction events since stimulation with high K^+^ of the same duration as that with IBMX did not show any alteration in firing activity in WT and KO OSNs.

During IBMX stimulation, while both WT and KO neurons showed spike trains with action potentials that were decreased in size, only the KO neurons fired few action potentials (a shorter spike train; Fig. 5).

Is the odorant response also modulated by STOML3? To address this question, we stimulated OSNs for 3 s with a mix of odorants (Fig. 5*F*,*G*) and recorded the evoked firing activity. As with IBMX stimulation, WT and KO OSNs both responded with a series of action potentials of decreasing size and at the same frequency (Fig. 5*J*). As with IBMX, clear differences emerged when we counted the number of spikes and the duration of the response, which were significantly lower and shorter in the KO neurons than in the WT neurons (3.5 ± 0.3 for KO, and 7 ± 1 for WT, *p* < 0.01 *U* test; 95 ± 18 ms for KO, 189 ± 34 ms for WT, *p* < 0.01 *U* test; Fig. 5*H*,*I*).

In summary, the differences in evoked firing activity in response to IBMX and the odorant mix compared with high K^+^ stimulation indicate that STOML3 functionally participates in transduction events.

## Discussion

In this study, we provide insights into the functional role of the STOML3 protein in OSNs. It was shown to be expressed in mouse OSNs (Kobayakawa et al., 2002; Goldstein et al., 2003; Kulaga et al., 2004; Tadenev et al., 2011), and our results confirmed the knob/proximal ciliary localization of STOML3 (Fig. 1). We also determined that the loss of STOML3 does not alter the morphology and cellular composition of the olfactory epithelium (Fig. 3). Interestingly, STOML3 has been shown to be mislocalized in BBS8 KO mice (Tadenev et al., 2011). BBS proteins are involved in ciliogenesis and ciliary transport (Jin and Nachury, 2009; Nachury and Mick, 2019), so we tested whether STOML3 might participate in these processes as well. We did not observe any significant alteration in the organization of the transduction machinery (Fig. 2) or in the ciliary structure (Fig. 3) of *Stoml3* KO mice, thus we conclude that STOML3 is not involved in the regulation of the olfactory epithelium structural and morphologic properties. However, we found evidence that STOML3 might have a functional role regulating the odorant transduction events.

Using only biochemical methods, previous studies have shown that STOML3 interacts with ACIII (Kobayakawa et al., 2002). Although these methods have traditionally been used to understand signal transduction in OSNs (Schreiber et al., 2000; Wei et al., 2004), they lack the resolution of electrophysiological techniques. To better understand the functional role of STOML3 in OSNs, we used loose patch recordings and found that spontaneous activity is altered when STOML3 is missing, indicating a potential role of STOML3 in modulating spontaneous action potential firing (Fig. 4). In OSNs, it has been shown that spontaneous firing is dependent on the activation of the signal transduction machinery by the constitutive activity of ORs (Reisert, 2010; Connelly et al., 2013). OR basal activation leads to basal cAMP oscillations that trigger the opening of CNG channels followed by that of TMEM16B. The resulting depolarization allows the OSN to fire action potentials that could be suppressed by blocking TMEM16B activity or knocking down other elements of transduction (Cygnar and Zhao, 2009; Reisert, 2010; Dibattista and Reisert, 2016; Pietra et al., 2016). Our experiments excluded the possibility that a significant alteration in the voltage-gated channels could be responsible for the observed alterations in firing behavior. Because of the STOML3 expression site and the origin of spontaneous activity in OSNs, we could speculate that STOML3 might be a new player in olfactory transduction.

In addition, we found that OSNs lacking STOML3 responded to IBMX stimulation with shorter spike trains than WT OSNs from P0/P4 mice (Fig. 5). The spike trains in the KO OSNs rapidly shut off and did not reappear during the duration of the entire stimulus. The same effects were observed during stimulation with the odor mixture (Fig. 5) but not during the application of the high K^+^ solution, again strengthening our indications that STOML3 is a modulator of transduction events. However, we cannot rule out the possibility that STOML3 could modulate neuronal excitability without being directly involved in the transduction events.

We could envision at least three possible scenarios for the role of STOML3 in olfactory transduction.

First, since spontaneous firing is driven by OR constitutive activity, STOML3 could modulate OR expression or transport of the OR to the ciliary site. Indeed, stomatin, another protein of the same family that also contains the stomatin motif, has been shown previously to interact with GPCRs (Wang et al., 2014; Park et al., 2016), thus making it possible that STOML3 might also function similarly in OSNs. In particular, it could regulate OR assembly on the ciliary membrane by mitigating the translocation of ORs with medium/high basal activity to keep basal noise instructive without altering the signal-to-noise ratio in OSNs (Nakashima et al., 2019). Indeed, a higher basal rate of constitutive activity could result in an increased cAMP production rate that would likely keep the cell in a more adapted state, thus limiting action potential firing (as we observed in *Stoml3* KO neurons).

The second scenario is based on the possible functional interaction of STOML3 with ACIII. Immunoprecipitation assays showed that STOML3 physically interacts with ACIII and that it is involved in cAMP production. In particular, using antibodies raised against STOML3, it was shown that in ciliary preparations, the cAMP concentration was increased compared with the control when ACIII was stimulated with forskolin (Kobayakawa et al., 2002). In the absence of STOML3, continuous cAMP production by the transduction cascade could lead to OSNs being in an adapted state (Reisert and Matthews, 1999; Reisert et al., 2007; Dibattista and Reisert, 2016), slowing down transduction events by reducing the chance of both spontaneous and evoked action potential firing. In addition, the differences in responses to IBMX between WT and KO neurons could strengthen this hypothesis, as they suggest that STOML3 might participate in the poorly understood cAMP buffering system of OSN cilia. Indeed, STOML3, by simply anchoring to the ciliary membrane in close proximity to ACIII, could buffer cAMP, keeping it relatively low in close proximity to the CNG channels or PDEs.

The third scenario we propose is a possible interaction between STOML3 and TMEM16B channels, similar to the STOML3 interaction with ASIC2a, ASIC2b, and ASIC3 (Price et al., 2004; Wetzel et al., 2007; Lapatsina et al., 2012; Klipp et al., 2020). STOML3 also functionally interacts with the mechanosensitive ion channels Piezo-1 and Piezo-2, increasing the sensitivity of these channels to mechanical stimulation (Poole et al., 2014). It would then be tempting to speculate a possible modulation of TMEM16B by STOML3. It has been previously shown that TMEM16B is an important regulator of the spontaneous and evoked firing of OSNs. TMEM16B could both amplify and “clamp” the resulting depolarization following the activation of signal transduction events in spontaneous and evoked firing, respectively. In the former, STOML3 could modulate the TMEM16B current by fastening its kinetics so that it could operate to amplify the smaller spontaneous events without causing sustained depolarization that would then terminate the firing, thus altering the spontaneous firing rate of OSNs. The same mechanism could account for the “clamping” effect of TMEM16B during evoked firing. When STOML3 was knocked out, the resulting slower TMEM16B kinetics could account for the persistent depolarization that would maintain the inactivation of voltage-gated channels (Trotier, 1994), allowing for fewer spikes to be generated over a shorter time window.

Additional experiments are needed to elucidate which of the proposed scenarios could occur in OSNs. Based on our results, we conclude that STOML3 is expressed in the knob and proximal ciliary regions in the olfactory epithelium and, for the first time, we showed that it participates in signal transduction events at least by regulating their output: action potential firing. However, since we found a sparse and punctate localization of STOML3 in the somato-dendritic regions, it could be that, like OMP (Dibattista et al., 2021), STOML3 have different functions in different OSN’s compartments. It is worth noting that recently emerging new players have been proposed to be involved in odorant transduction mechanisms (Buiakova et al., 1996; Ivic et al., 2000; Sinnarajah et al., 2001; Von Dannecker et al., 2005; Kerr et al., 2008; Dooley et al., 2009; Kaneko-Goto et al., 2013; Baumgart et al., 2014; Dibattista and Reisert, 2016; Talaga et al., 2017), increasing the complexity of long-known mechanisms crucial for the transformation of a chemical signal, the odorant, into an electrical signal, the action potential, that could then initiate the long journey to the brain.

## Synthesis

Reviewing Editor: Christina Zelano, Northwestern University Decisions are customarily a result of the Reviewing Editor and the peer reviewers coming together and discussing their recommendations until a consensus is reached. When revisions are invited, a fact-based synthesis statement explaining their decision and outlining what is needed to prepare a revision will be listed below. The following reviewer(s) agreed to reveal their identity: Stephan Frings.

The reviewers were both enthusiastic about the strength and importance of this work. We all agreed that the findings are exciting, and begin to fill an important gap in the field. We all agreed that only minor changes are needed prior to publication of this interesting manuscript.

Changes needed:

- Differences between wt an ko neurons occurred upon application of IBMX and odorants, but not in response to elevated potassium, an observation that the authors present as evidence for a specific role of STOML3 within the transduction pathway - as opposed to an effect on action potential generation. This central point of their story is not supported by clear evidence, as STOML3 appears to be expressed both in the ciliary knobs (transduction) and in the somato-dendritic region (AP generation). Moreover, the stimuli - IBMX and odorants - were also applied to both regions, so that the STOML3 effect cannot be localized to the ciliary region with any certainty. This is an important point as it underlines the complete absence of any mechanistic evidence for how STOML3 may promote excitability in these neurons. Nevertheless, the auhors did a good job in offering in the Discussion three scenarios for a possible role of STOML3, three concepts that certainly will aid future work.

In the manuscript, the ambiguity concerning the exact site of STOML3 action can be dealt with by appropriate wording. It does not diminish the significance of the main finding, the necessity to have STOML3 in olfactory receptor neurons to achieve full excitability. This is indeed very interesting and, possibly, important for olfactory physiology.

Other minor comments can be found below, in the reviewers original comments:

Reviewer 1:

This is a straightforward study using a knockout mouse model to investigate Stomatin-like protein-3 (STOML3) functional relevance in olfactory sensory neurons (OSNs). STOML3 has been identified as an OSN specific protein for almost 20 years. Its role in olfactory sensory transduction has been postulated based on its cilial localization. This physiological role, however, has never been further addressed. Whether STOML3 may have other functional roles in OSNs are also not known. In this manuscript, the authors first confirmed the cilial and apical dendritic localization of STOML3 in olfactory sensory neurons using immunofluorescence. They then characterized the olfactory epithelium of Stoml3 knockout mice and reported no alteration in general morphology, cellular composition, expression of several olfactory transduction components, and OSN cilia. The authors then investigated both spontaneous and evoked firing of action potentials in Stoml3 knockout OSNs, by single-cell loose-patch recording from olfactory epithelial slices of P0-P4 pups and showed that the knockout OSNs displayed reduced spontaneous firing frequency and reduced firing duration and spike numbers to odor and the PDE blocker IBMX. The authors also investigated high K+-induced firing in OSNs and found no difference between the wildtype and the knockout. The author thus demonstrated a role of STOML3 in modulating olfactory sensory transduction.

This reviewer finds that the experiments described in the manuscript are well conducted. The methods used are appropriate. Although spike firing is not a direct measure of the transduction process, the high K+ experiment allows the authors to justify that STOML3 acts at the olfactory transduction level. The results are clear and convincing. The conclusion is valid. Demonstrating that STOML3 functions as a modulator of olfactory transduction is significant. The manuscript leaves many unanswered questions to the future, including the mechanism underlying STOML3’s modulatory role in olfactory transduction and the behavioral significance of this modulation. I have no major concerns but a couple of minor comments.

Minor comments:

1. Figure 2B, D, F, H, J, and L are redundant with Figure 1A and D, at the presented resolution. The authors can consider either delete these figures in Figure 2 or present high magnification zoom-ins for colocalization.

2. Although it is described in the Method, it is still better to clearly state in Results and/or in Discussion that the electrophysiological recordings are done from OSNs of P0-P4 pups.

Reviewer 2:

Current understanding of how signal transduction in olfactory receptor neurons works has reached a level where multiple modulatory processes became evident, processes that support, change, or terminate the canonical transduction cascade. Modulatory effects were reported for odor receptors, for GTP-binding proteins, for cAMP synthesis, for ion channels in the chemosensory cilia, and these effects clearly shape the afferent signals before they leave the nose. In the present manuscript, the authors provide evidence that the protein STOML3, which has been a candidate for such a modulatory function for some time, has a measurable effect on the excitability of mouse olfactory receptor neurons. A comparison of wildtype and STOML3 knockout animals points to a functional role of this protein in supporting both spontaneous activity and the response to odorants.

Differences between wt an ko neurons occurred upon application of IBMX and odorants, but not in response to elevated potassium, an observation that the authors present as evidence for a specific role of STOML3 within the transduction pathway - as opposed to an effect on action potential generation. This central point of their story is not supported by clear evidence, as STOML3 appears to be expressed both in the ciliary knobs (transduction) and in the somato-dendritic region (AP generation). Moreover, the stimuli - IBMX and odorants - were also applied to both regions, so that the STOML3 effect cannot be localized to the ciliary region with any certainty. I think this is an important point as it underlines the complete absence of any mechanistic evidence for how STOML3 may promote excitability in these neurons. Nevertheless, the auhors did a good job in offering in the Discussion three scenarios for a possible role of STOML3, three concepts that certainly will aid future work.

In the manuscript, the ambiguity concerning the exact site of STOML3 action can be dealt with by appropriate wording. It does not diminish the significance of the main finding, the necessity to have STOML3 in olfactory receptor neurons to achieve full excitability. This is indeed very interesting and, possibly, important for olfactory physiology.

In revising the mansucript I also would suggest to address the following points:

1. lines 152-156: Explain criteria for the identification of proteins in WB. Include protein size. Were the proteins deglycosylated? Some look like it.

2. lines 188-191/ 283: Include these wh.c. control experiments in the result section. Show statistics.

3. lines 192-196: Justify the use of 5 × 100 μM odorants and 1 mM IBMX.

4. lines 111 & 266: Explain for the reader what you mean by "area". How does this term correspond to the number/ density of cells in the tissue?

5. lines 290-293: How does the variance of firing rates and ISIs compare to published data? Are they OK, or do they result from problems with your preparation?

## References

[B1] Arnson HA, Holy TE (2011) Chemosensory burst coding by mouse vomeronasal sensory neurons. J Neurophysiol 106:409–420. 10.1152/jn.00108.2011 21525370 PMC3129729

[B2] Baumgart S, Jansen F, Bintig W, Kalbe B, Herrmann C, Klumpers F, Köster SD, Scholz P, Rasche S, Dooley R, Metzler-Nolte N, Spehr M, Hatt H, Neuhaus EM (2014) The scaffold protein MUPP1 regulates odorant-mediated signaling in olfactory sensory neurons. J Cell Sci 127:2518–2527. 10.1242/jcs.144220 24652834

[B3] Billig GM, Pál B, Fidzinski P, Jentsch TJ (2011) Ca2+-activated Cl− currents are dispensable for olfaction. Nat Neurosci 14:763–769. 10.1038/nn.2821 21516098

[B4] Brand J, Smith ESJ, Schwefel D, Lapatsina L, Poole K, Omerbašić D, Kozlenkov A, Behlke J, Lewin GR, Daumke O (2012) A stomatin dimer modulates the activity of acid-sensing ion channels. EMBO J 31:3635–3646. 10.1038/emboj.2012.203 22850675 PMC3433786

[B5] Browman DT, Hoegg MB, Robbins SM (2007) The SPFH domain-containing proteins: more than lipid raft markers. Trends Cell Biol 17:394–402. 10.1016/j.tcb.2007.06.005 17766116

[B6] Brunet LJ, Gold GH, Ngai J (1996) General anosmia caused by a targeted disruption of the mouse olfactory cyclic nucleotide-gated cation channel. Neuron 17:681–693. 10.1016/s0896-6273(00)80200-7 8893025

[B7] Buck L, Axel R (1991) A novel multigene family may encode odorant receptors: a molecular basis for odor recognition. Cell 65:175–187. 10.1016/0092-8674(91)90418-x 1840504

[B8] Buiakova OI, Baker H, Scott JW, Farbman A, Kream R, Grillo M, Franzen L, Richman M, Davis LM, Abbondanzo S, Stewart CL, Margolis FL (1996) Olfactory marker protein (OMP) gene deletion causes altered physiological activity of olfactory sensory neurons. Proc Natl Acad Sci USA 93:9858–9863. 10.1073/pnas.93.18.9858 8790421 PMC38519

[B9] Connelly T, Savigner A, Ma M (2013) Spontaneous and sensory-evoked activity in mouse olfactory sensory neurons with defined odorant receptors. J Neurophysiol 110:55–62. 10.1152/jn.00910.2012 23596334 PMC3727041

[B10] Cygnar KD, Zhao H (2009) Phosphodiesterase 1C is dispensable for rapid response termination of olfactory sensory neurons. Nat Neurosci 12:454–462. 10.1038/nn.2289 19305400 PMC2712288

[B11] Dibattista M, Reisert J (2016) The odorant receptor-dependent role of olfactory marker protein in olfactory receptor neurons. J Neurosci 36:2995–3006. 10.1523/JNEUROSCI.4209-15.2016 26961953 PMC4783500

[B12] Dibattista M, Mazzatenta A, Grassi F, Tirindelli R, Menini A (2008) Hyperpolarization-activated cyclic nucleotide-gated channels in mouse vomeronasal sensory neurons. J Neurophysiol 100:576–586. 10.1152/jn.90263.2008 18509074

[B13] Dibattista M, Pifferi S, Boccaccio A, Menini A, Reisert J (2017) The long tale of the calcium activated Cl- channels in olfactory transduction. Channels (Austin) 11:399–414. 10.1080/19336950.2017.1307489 28301269 PMC5626357

[B14] Dibattista M, Al Koborssy D, Genovese F, Reisert J (2021) The functional relevance of olfactory marker protein in the vertebrate olfactory system: a never-ending story. Cell Tissue Res 383:409–427. 10.1007/s00441-020-03349-9 33447880 PMC7878404

[B15] Dooley R, Baumgart S, Rasche S, Hatt H, Neuhaus EM (2009) Olfactory receptor signaling is regulated by the post-synaptic density 95, Drosophila discs large, zona-occludens 1 (PDZ) scaffold multi-PDZ domain protein 1. FEBS J 276:7279–7290. 10.1111/j.1742-4658.2009.07435.x 19909339

[B16] Firestein S (2001) How the olfactory system makes sense of scents. Nature 413:211–218. 10.1038/35093026 11557990

[B17] Goldstein BJ, Kulaga HM, Reed RR (2003) Cloning and characterization of SLP3: a novel member of the stomatin family expressed by olfactory receptor neurons. J Assoc Res Otolaryngol JARO 4:74–82. 10.1007/s10162-002-2039-5 12239636 PMC3202447

[B18] Green JB, Young JPW (2008) Slipins: ancient origin, duplication and diversification of the stomatin protein family. BMC Evol Biol 8:44. 10.1186/1471-2148-8-44 18267007 PMC2258279

[B19] Henriques T, Agostinelli E, Hernandez-Clavijo A, Maurya DK, Rock JR, Harfe BD, Menini A, Pifferi S (2019) TMEM16A calcium-activated chloride currents in supporting cells of the mouse olfactory epithelium. J Gen Physiol 151:954–966. 10.1085/jgp.201812310 31048412 PMC6605691

[B20] Hunyady B, Krempels K, Harta G, Mezey E (1996) Immunohistochemical signal amplification by catalyzed reporter deposition and its application in double immunostaining. J Histochem Cytochem 44:1353–1362. 10.1177/44.12.8985127 8985127

[B21] Ivic L, Pyrski MM, Margolis JW, Richards LJ, Firestein S, Margolis FL (2000) Adenoviral vector-mediated rescue of the OMP-null phenotype in vivo. Nat Neurosci 3:1113–1120. 10.1038/80632 11036268

[B91] Jin H, Nachury MV (2009) The BBsome. Curr Biopl 19:R472-3. 10.1152/jn.00108.2011 19549489

[B22] Jones DT, Reed RR (1989) Golf: an olfactory neuron specific-G protein involved in odorant signal transduction. Science 244:790–795. 10.1126/science.2499043 2499043

[B23] Kaneko-Goto T, Sato Y, Katada S, Kinameri E, Yoshihara S, Nishiyori A, Kimura M, Fujita H, Touhara K, Reed RR, Yoshihara Y (2013) Goofy coordinates the acuity of olfactory signaling. J Neurosci 33:12987–12996, 12996a. 10.1523/JNEUROSCI.4948-12.2013 23926254 PMC6619734

[B24] Kerr DS, Von Dannecker LEC, Davalos M, Michaloski JS, Malnic B (2008) Ric-8B interacts with G alpha olf and G gamma 13 and co-localizes with G alpha olf, G beta 1 and G gamma 13 in the cilia of olfactory sensory neurons. Mol Cell Neurosci 38:341–348. 10.1016/j.mcn.2008.03.006 18462949

[B25] Kleene SJ (2008) The electrochemical basis of odor transduction in vertebrate olfactory cilia. Chem Senses 33:839–859. 10.1093/chemse/bjn048 18703537

[B26] Klipp RC, Cullinan MM, Bankston JR (2020) Insights into the molecular mechanisms underlying the inhibition of acid-sensing ion channel 3 gating by stomatin. J Gen Physiol 152:e201912471. 32012213 10.1085/jgp.201912471 PMC7054857

[B27] Kobayakawa K, Hayashi R, Morita K, Miyamichi K, Oka Y, Tsuboi A, Sakano H (2002) Stomatin-related olfactory protein, SRO, specifically expressed in the murine olfactory sensory neurons. J Neurosci 22:5931–5937. 10.1523/JNEUROSCI.22-14-05931.2002 12122055 PMC6757947

[B28] Kulaga HM, Leitch CC, Eichers ER, Badano JL, Lesemann A, Hoskins BE, Lupski JR, Beales PL, Reed RR, Katsanis N (2004) Loss of BBS proteins causes anosmia in humans and defects in olfactory cilia structure and function in the mouse. Nat Genet 36:994–998. 10.1038/ng1418 15322545

[B29] Lapatsina L, Brand J, Poole K, Daumke O, Lewin GR (2012) Stomatin-domain proteins. Eur J Cell Biol 91:240–245. 10.1016/j.ejcb.2011.01.018 21501885

[B30] Lipscomb BW, Treloar HB, Greer CA (2002) Cell surface carbohydrates reveal heterogeneity in olfactory receptor cell axons in the mouse. Cell Tissue Res 308:7–17. 10.1007/s00441-002-0532-0 12012202

[B31] Lorenzon P, Redolfi N, Podolsky MJ, Zamparo I, Franchi SA, Pietra G, Boccaccio A, Menini A, Murthy VN, Lodovichi C (2015) Circuit formation and function in the olfactory bulb of mice with reduced spontaneous afferent activity. J Neurosci 35:146–160. 10.1523/JNEUROSCI.0613-14.2015 25568110 PMC6605243

[B32] Mombaerts P (2004) Genes and ligands for odorant, vomeronasal and taste receptors. Nat Rev Neurosci 5:263–278. 10.1038/nrn1365 15034552

[B92] Nachury MV, Mick DU (2019) Establishing and regulating the composition of cilia for signal transduction. Nat Rev Mol Cell Biol 20:389–405. 30948801 10.1038/s41580-019-0116-4 PMC6738346

[B33] Nakashima A, Ihara N, Shigeta M, Kiyonari H, Ikegaya Y, Takeuchi H (2019) Structured spike series specify gene expression patterns for olfactory circuit formation. Science 365:eaaw5030. 10.1126/science.aaw5030 31171707

[B34] Park MY, Kim N, Wu LL, Yu GY, Park K (2016) Role of flotillins in the endocytosis of GPCR in salivary gland epithelial cells. Biochem Biophys Res Commun 476:237–244. 10.1016/j.bbrc.2016.05.103 27221048

[B35] Pietra G, Dibattista M, Menini A, Reisert J, Boccaccio A (2016) The Ca2+-activated Cl- channel TMEM16B regulates action potential firing and axonal targeting in olfactory sensory neurons. J Gen Physiol 148:293–311. 10.1085/jgp.201611622 27619419 PMC5037344

[B36] Pifferi S, Menini A, Kurahashi T (2010) Signal transduction in vertebrate olfactory cilia. In: The neurobiology of olfaction, frontiers in neuroscience (Menini A, ed). Boca Raton: CRC/Taylor and Francis. 21882437

[B37] Poole K, Herget R, Lapatsina L, Ngo HD, Lewin GR (2014) Tuning Piezo ion channels to detect molecular-scale movements relevant for fine touch. Nat Commun 5:3520. 10.1038/ncomms4520 24662763 PMC3973071

[B38] Potter SM, Zheng C, Koos DS, Feinstein P, Fraser SE, Mombaerts P (2001) Structure and emergence of specific olfactory glomeruli in the mouse. J Neurosci 21:9713–9723. 10.1523/JNEUROSCI.21-24-09713.2001 11739580 PMC2570017

[B39] Price MP, Thompson RJ, Eshcol JO, Wemmie JA, Benson CJ (2004) Stomatin modulates gating of acid-sensing ion channels. J Biol Chem 279:53886–53891. 10.1074/jbc.M407708200 15471860

[B40] Qi Y, Andolfi L, Frattini F, Mayer F, Lazzarino M, Hu J (2015) Membrane stiffening by STOML3 facilitates mechanosensation in sensory neurons. Nat Commun 6:8512. 10.1038/ncomms9512 26443885 PMC4633829

[B41] Reisert J (2010) Origin of basal activity in mammalian olfactory receptor neurons. J Gen Physiol 136:529–540. 10.1085/jgp.201010528 20974772 PMC2964517

[B42] Reisert J, Matthews HR (1999) Adaptation of the odour-induced response in frog olfactory receptor cells. J Physiol 519:801–813. 10.1111/j.1469-7793.1999.0801n.x 10457092 PMC2269541

[B43] Reisert J, Reingruber J (2019) Ca2+-activated Cl- current ensures robust and reliable signal amplification in vertebrate olfactory receptor neurons. Proc Natl Acad Sci USA 116:1053–1058. 10.1073/pnas.1816371116 30598447 PMC6338846

[B44] Reisert J, Yau KW, Margolis FL (2007) Olfactory marker protein modulates the cAMP kinetics of the odour-induced response in cilia of mouse olfactory receptor neurons. J Physiol 585:731–740. 10.1113/jphysiol.2007.142471 17932148 PMC2375515

[B45] Schild D, Restrepo D (1998) Transduction mechanisms in vertebrate olfactory receptor cells. Physiol Rev 78:429–466. 10.1152/physrev.1998.78.2.429 9562035

[B46] Schindelin J, Arganda-Carreras I, Frise E, Kaynig V, Longair M, Pietzsch T, Preibisch S, Rueden C, Saalfeld S, Schmid B, Tinevez J-Y, White DJ, Hartenstein V, Eliceiri K, Tomancak P, Cardona A (2012) Fiji: an open-source platform for biological-image analysis. Nat Methods 9:676–682. 10.1038/nmeth.2019 22743772 PMC3855844

[B47] Schreiber S, Fleischer J, Breer H, Boekhoff I (2000) A possible role for caveolin as a signaling organizer in olfactory sensory membranes. J Biol Chem 275:24115–24123. 10.1074/jbc.M001876200 10816570

[B48] Shimazaki R, Boccaccio A, Mazzatenta A, Pinato G, Migliore M, Menini A (2006) Electrophysiological properties and modeling of murine vomeronasal sensory neurons in acute slice preparations. Chem Senses 31:425–435. 10.1093/chemse/bjj047 16547196

[B49] Sinnarajah S, Dessauer CW, Srikumar D, Chen J, Yuen J, Yilma S, Dennis JC, Morrison EE, Vodyanoy V, Kehrl JH (2001) RGS2 regulates signal transduction in olfactory neurons by attenuating activation of adenylyl cyclase III. Nature 409:1051–1055. 10.1038/35059104 11234015

[B50] Tadenev ALD, Kulaga HM, May-Simera HL, Kelley MW, Katsanis N, Reed RR (2011) Loss of Bardet-Biedl syndrome protein-8 (BBS8) perturbs olfactory function, protein localization, and axon targeting. Proc Natl Acad Sci USA 108:10320–10325. 10.1073/pnas.1016531108 21646512 PMC3121838

[B51] Talaga AK, Dong FN, Reisert J, Zhao H (2017) Cilia- and flagella-associated protein 69 regulates olfactory transduction kinetics in mice. J Neurosci 37:5699–5710. 10.1523/JNEUROSCI.0392-17.2017 28495971 PMC5469307

[B52] Tavernarakis N, Driscoll M, Kyrpides NC (1999) The SPFH domain: implicated in regulating targeted protein turnover in stomatins and other membrane-associated proteins. Trends Biochem Sci 24:425–427. 10.1016/s0968-0004(99)01467-x 10542406

[B53] Trotier D (1994) Intensity coding in olfactory receptor cells. Semin Cell Biol 5:47–54. 10.1006/scel.1994.1007 7514456

[B54] Umlauf E, Mairhofer M, Prohaska R (2006) Characterization of the stomatin domain involved in homo-oligomerization and lipid raft association. J Biol Chem 281:23349–23356. 10.1074/jbc.M513720200 16766530

[B55] Von Dannecker LEC, Mercadante AF, Malnic B (2005) Ric-8B, an olfactory putative GTP exchange factor, amplifies signal transduction through the olfactory-specific G-protein Galphaolf. J Neurosci 25:3793–3800. 10.1523/JNEUROSCI.4595-04.2005 15829631 PMC6724935

[B56] Wang YJ, Guo XL, Li SA, Zhao YQ, Liu ZC, Lee WH, Xiang Y, Zhang Y (2014) Prohibitin is involved in the activated internalization and degradation of protease-activated receptor 1. Biochim Biophys Acta 1843:1393–1401. 10.1016/j.bbamcr.2014.04.005 24732013

[B57] Wei CJ, Xu X, Lo CW (2004) Connexins and cell signaling in development and disease. Annu Rev Cell Dev Biol 20:811–838. 10.1146/annurev.cellbio.19.111301.144309 15473861

[B58] Wetzel C, Hu J, Riethmacher D, Benckendorff A, Harder L, Eilers A, Moshourab R, Kozlenkov A, Labuz D, Caspani O, Erdmann B, Machelska H, Heppenstall PA, Lewin GR (2007) A stomatin-domain protein essential for touch sensation in the mouse. Nature 445:206–209. 10.1038/nature05394 17167420

[B59] Wetzel C, Pifferi S, Picci C, Gök C, Hoffmann D, Bali KK, Lampe A, Lapatsina L, Fleischer R, Smith ES, Bégay V, Moroni M, Estebanez L, Kühnemund J, Walcher J, Specker E, Neuenschwander M, von Kries JP, Haucke V, Kuner R, et al. (2017) Small-molecule inhibition of STOML3 oligomerization reverses pathological mechanical hypersensitivity. Nat Neurosci 20:209–218. 10.1038/nn.4454 27941788

[B60] Wong ST, Trinh K, Hacker B, Chan GC, Lowe G, Gaggar A, Xia Z, Gold GH, Storm DR (2000) Disruption of the type III adenylyl cyclase gene leads to peripheral and behavioral anosmia in transgenic mice. Neuron 27:487–497. 10.1016/s0896-6273(00)00060-x 11055432

[B61] Wong WM, Nagel M, Hernandez-Clavijo A, Pifferi S, Menini A, Spehr M, Meeks JP (2018) Sensory adaptation to chemical cues by vomeronasal sensory neurons. eNeuro 5:ENEURO.0223-18.2018. 10.1523/ENEURO.0223-18.2018 PMC6088365 30105301

[B62] Zhang Y, Zhang Z, Xiao S, Tien J, Le S, Le T, Jan LJ, Yang H (2017) Inferior olivary TMEM16B mediates cerebellar motor learning. Neuron 95:1103–1111. 10.1016/j.neuron.2017.08.010 28858616 PMC5659299

